# Total anti-SARS-CoV-2 antibodies measured 6 months after Pfizer-BioNTech COVID-19 vaccination in healthcare workers

**DOI:** 10.5937/jomb0-33999

**Published:** 2022-04-08

**Authors:** Gian Luca Salvagno, Brandon M. Henry, Laura Pighi, Nitto Simone de, Giuseppe Lippi

**Affiliations:** 1 University of Verona, Section of Clinical Biochemistry, Verona, Italy; 2 Pederzoli Hospital, Service of Laboratory Medicine, Peschiera del Garda, Italy; 3 Cincinnati Children's Hospital Medical Center, The Heart Institute, Cardiac Intensive Care Unit, Cincinnati, Ohio, USA; 4 Texas Biomedical Research Institute, Host-Pathogen Interactions & Population Health Programs, San Antonio, Texas, USA

**Keywords:** COVID-19, SARS-CoV-2, vaccination, antibodies, immune response, COVID-19, SARS-CoV-2, vakcinacija, antitela, imuni odgovor

## Abstract

**Background:**

This study aimed at monitoring the kinetics of serum total anti-SARS-CoV-2 (severe acute respiratory syndrome coronavirus 2) antibodies in a cohort of healthcare workers after voluntary vaccination with Pfizer-BioNTech coronavirus disease 2019 (COVID-19) mRNA-based vaccine.

**Methods:**

The study population consisted of 787 healthcare workers (mean age 44±12 years; 66% females), who received two 30 mg doses of Pfizer-BioNTech COVID-19 vaccine, 3 weeks apart. Venous blood was drawn before the first vaccine dose, immediately before the second vaccine dose, and then at 1, 3 and 6 months after the second vaccine dose. Serological testing employed the total antiSARS-CoV-2 antibodies measurement with Roche Elecsys Anti-SARS-CoV-2 S chemiluminescent immunoassay.

**Results:**

The median serum levels of total anti-SARS-CoV-2 antibodies reached the peak (1762 kU/L) 1 month after the second vaccine dose, but tended to progressively decline at the 3-month (1086 kU/L) and 6-month (802 kU/L) follow-up points. Overall, the values after 3and 6months were 37% and 57% lower than the corresponding concentrations measured at the peak. No healthcare worker had total anti-SARS-CoV-2 antibodies below the method-dependent cut-off after 6 months. The decline compared to the peak was more accentuated in baseline seropositive persons than in those who were baseline seronegative (74% vs. 52%) cohort. The 6-month post-vaccination anti-SARS-CoV-2 antibodies in subjects aged <65 years remained over 2-fold higher than in those aged ≥65 years (813 vs. 343 kU/L) and also remained consistently higher in women than in men.

**Conclusions:**

Gradual decline of total anti-SARS-CoV-2 antibodies occurred 6 months after Pfizer-BioNTech COVID-19 vaccination, though values remained higher than the method-dependent cut-off, with no case of sero-negativization.

## Abstract (sr)

**Uvod:**

Ova studija je imala za cilj praćenje kinetike ukupnih serumskih antitela na SARS-CoV-2 (teški akutni respiratorni sindrom koronavirus 2) u kohorti zdravstvenih radnika nakon dobrovoljne vakcinacije sa Pfizer-BioNTech koronavirusnom bolešću 2019 (COVID-19) na bazi mRNA-vakcine.

**Metode:**

Ispitivanu populaciju činilo je 787 zdravstvenih radnika (prosečna starost 44 ± 12 godina; 66% žena), koji su primili dve doze od 30 mg vakcine Pfizer-BioNTech protiv COVID-19, u razmaku od 3 nedelje. Venska krv je uzeta pre prve doze vakcine, neposredno pre druge doze vakcine, a zatim 1, 3 i 6 meseci nakon druge doze vakcine. Serološko testiranje je koristilo merenje ukupnih anti-SARS-CoV-2 antitela pomoću Roche Elecsis Anti-SARS-CoV-2 S hemiluminiscentnog imunološkog testa.

**Rezultati:**

Srednji serumski nivo ukupnih antitela na SARSCoV-2 dostigao je vrhunac (1762 kU/L) 1 mesec nakon druge doze vakcine, ali je imao tendenciju progresivnog opadanja tokom 3 meseca (1086 kU/L) i 6-mesečni (802 kU/L) praćenja. Sve u svemu, vrednosti nakon 3 i 6 meseci bile su 37% i 57% niže od odgovarajućih koncentracija izmerenih na vrhuncu. Nijedan zdravstveni radnik nije imao ukupna antitela na SARS-CoV-2 ispod granične vrednosti zavisno od metode. Pad u odnosu na vrhunac bio je više naglašen kod osnovnih seropozitivnih osoba nego kod onih koje su bile osnovno seronegativne (74% naspram 52%) kohorte. Šestomesečna post-vakcinalna antitela na SARS-CoV-2 kod ispitanika mla|ih od 65 godina ostala su preko dva puta veća od onih izmerenih kod osoba starijih od 65 godina (813 naspram 343 kU/L) i tako|e su konstantno veća kod žena nego kod muškaraca.

**Zaključak:**

Postepeno opadanje ukupnih antitela na SARSCoV-2 dogodilo se 6 meseci nakon vakcinacije protiv PfizerBioNTech-a protiv COVID-19, iako su vrednosti ostale veće od preseka zavisnog od metode, bez slučaja seronegativi - zacije.

## Introduction

There is now consolidated evidence that the onset of SARS-CoV-2 (severe acute respiratory syndrome coronavirus 2) infection in healthcare facilities is associated with an enhanced risk of morbidity and mortality, both in hospitalized patients as well as in healthcare workers. Therefore, regular SARS-CoV-2 diagnostic testing and coronavirus disease 2019 (COVID-19) vaccination are now regarded as major cornerstones for preventing and/or limiting the burden of SARS-CoV-2 inside and outside healthcare facilities [1]. A recent study published by Yoshimura et al. [2] evidenced a fairly good response in terms of anti-SARS-CoV-2 IgG level after a complete cycle of Pfizer BNT162b2 mRNA-based COVID-19 vaccine. Since it is now acknowledged that large part of vaccine effectiveness is attributable to the generation of anti-SARS-CoV-2 antibodies of different classes and with different antigenic targets [3], but capable to quickly and efficiently neutralize viral particles inside the host, regular assessment of these antibodies seems essential for monitoring immune protection among healthcare workers, especially given that serum levels of most vaccine-induced antibodies are observed to decline over time [4]. This study was hence aimed at monitoring the kinetics of serum total anti-SARS-CoV-2 antibodies in a cohort of healthcare workers who underwent voluntary administration of Pfizer-BioNTech COVID-19 mRNA-based vaccine.

## Materials and Methods

The study population consisted of 787 healthcare workers of Peschiera del Garda hospital in Italy (mean age 44±12 years; 66% females), who received two 30 mg doses of Pfizer-BioNTech COVID-19 vaccine, 3 weeks apart. Venous blood was drawn before the first vaccine dose, immediately before the second vaccine dose (i.e., 21 days after the first dose), and then at 1, 3 and 6 months after the second vaccine dose (51, 111 and 201 days after the first vaccine dose). Serological testing was based on total anti-SARS-CoV-2 antibodies measurement with Roche Elecsys Anti-SARS-CoV-2 S chemiluminescent immunoassay, on Roche Cobas 6000 (Roche Diagnostics, Basel, Switzerland; positive result: ≥0.8 kU/L). Recent evidence suggests that this method reliably mirrors the overall SARS-CoV-2 neutralizing potential developed after COVID-19 vaccination, with sensitivity as high as 98% compared to a pseudovirus neutralization test [5]. Test results were reported as median and interquartile range (IQR), and the statistical analysis was performed using Analyse-it (Analyse-it Software Ltd, Leeds, UK). Between-group comparisons were carried out with Mann-Whitney test. All study participants gave informed consents for vaccination and undergoing serial anti-SARS-CoV-2 antibodies monitoring. This observational study was reviewed and cleared by the Ethics Committee of Verona and Rovigo provinces (3246CESC).

## Results

**Figure 1:**
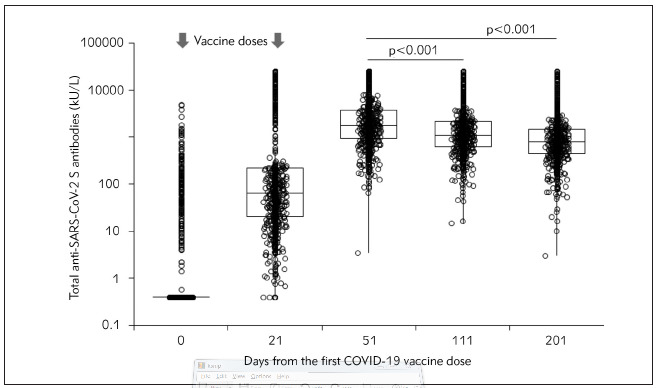
Kinetics of total anti-SARS-CoV-2 (severe acute respiratory syndrome coronavirus 2) serum antibodies in a cohort of healthcare workers who underwent voluntary administration of two doses of Pfizer-BioNTech COVID-19 mRNA-based vaccine

**Figure 2:**
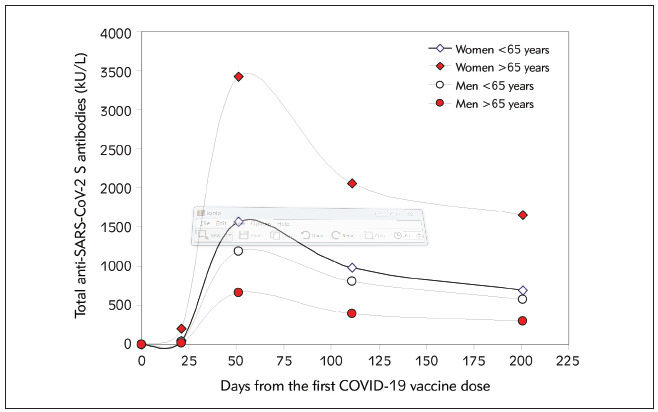
Kinetics of total anti-SARS-CoV-2 (severe acute respiratory syndrome coronavirus 2) serum antibodies in a cohort of healthcare workers who underwent voluntary administration of two doses of Pfizer-BioNTech COVID-19 mRNA-based vaccine, stratified by age and sex

**Figure 3:**
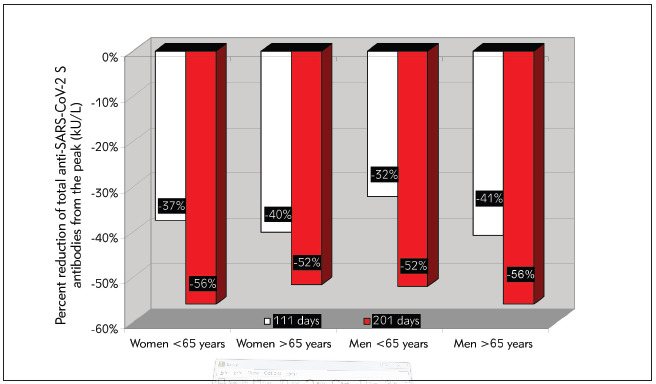
The relative decrease of the serum level of total anti-SARS-CoV-2 (severe acute respiratory syndrome coronavirus 2) antibodies compared to the peak after 3 and 6 months from coronavirus disease 2019 (COVID-19) vaccination, stratified by age and sex

The main results of this study are shown in Figure 1. The median serum levels of total anti-SARS-CoV-2 antibodies reached the peak (1762 kU/L; IQR, 933–3761 kU/L) 1 month after the second vaccine dose (i.e., 51 days after the first vaccine dose), but then tended to progressively decline at the 3-month (i.e., 111 days after the first vaccine dose: 1086 kU/L; IQR, 629–2155 kU/L) and 6-month (i.e., 201 days after the first vaccine dose: 802 kU/L; IQR, 447–1487 kU/L) follow-up points. Overall, the serum values after 3-and 6-months were 37% (IQR, 15–53%; p<0.001) and 57% (IQR, 35–71%; p<0.001) lower than the corresponding concentrations measured at the peak, respectively. Notably, no healthcare worker had total anti-SARS-CoV-2 antibodies below the method-dependent cut-off (i.e., 0.8 kU/L) at either the 3- or 6-month follow-up points. Notably, the relative decline of serum antibodies values compared to the peak was more accentuated in baseline seropositive (74%; IQR, 62–82%) persons than in those who were baseline seronegative (52%; IQR, 31–67%) cohort. A decrease of serum total anti-SARS-CoV-2 antibodies levels occurred in 576/624 (92.3%) baseline seronegative subjects and in 160/163 (98.1%) seropositive subjects. The 6-month post-vaccination serum level of total anti-SARS-CoV-2 antibodies in subjects aged <65 years (n=754; 813 KUL; IQR, 473-1501 kU/L) remained over 2-fold higher than that measured in those aged ≥65 years (n=33; 343 kU/L; IQR, 210-903 kU/L; p<0.001), and also remained consistently higher in women than in men (Figure 2). The relative decrease of the serum level of total anti-SARS-CoV-2 antibodies compared to the peak after 3 and 6 months from vaccination is also shown in Figure 3, where a very similar trend can be seen despite the rather different peak levels (Figure 3).

## Discussion

The preliminary results of this ad-interim analysis of anti-SARS-CoV-2 humoral immunity in healthcare workers who received a complete (2-dose) cycle of Pfizer-BioNTech COVID-19 vaccination reveal that a gradual decline of total anti-SARS-CoV-2 antibodies (and hence of neutralizing potential) has occ urred 6 months after vaccination, though the antibodies values have remained still considerably higher than the method-dependent cut-off and no seronegativization seems to have occurred in either baseline seronegative or seropositive subjects. These results compared rather well with other preliminary data made available by Bayart and colleagues [6], who also showed that the decay of anti-SARS-CoV-2 RBD total antibodies after 6 months from Pfizer-BioNTech COVID-19 vaccine administration was around 55% in seronegative healthcare workers, thus nearly identical to the 57% decrease that we found in our cohort over an identical period of monitoring. Although better medium-term response would have been theoretically expected in subjects with double immunization (i.e., SARS-CoV-2 infection followed by full vaccination), we instead found that total anti-SARS-CoV-2 antibodies reduction appeared larger in baseline seropositive than seronegative recipients (i.e., 74% vs. 52%) (Figure 2). This is perhaps attributable to the fact that the former cohort also displayed higher peak values, so that the catabolism may have been relatively more pronounced in these subjects. The difference in antibody levels distribution between age and sexes is not really unexpected, since some concomitant studies showed similar trends, with lower immunogenicity been reported in men and in the elderly [7] [8].

Notably, the significantly lower antibodies values found in elderly healthcare male workers (Figure 2) also raises further doubts as to whether further vaccine boosters may be especially advisable in this cohort between 6 and 12 months after completing a regular Pfizer-BioNTech COVID-19 vaccine cycle, to ensure a reinforced protection [9], since this is the most vulnerable population to severe SARS-CoV-2 infection [10].

## Dodatak

### Research funding

None declared.

### Author contributions

All authors have accepted responsibility for the entire content of this manuscript and approved its submission.

### Informed consent

Informed consent was obtained from all individuals included in this study.

### Ethical approval

This observational study was reviewed and cleared by the Ethics Committee of Verona and Rovigo provinces (3246CESC).

### Acknowledgments

The authors acknowledge the staff of the Service of Laboratory Medicine of the Pederzoli Hospital (Peschiera del Garda, Italy) for the skill technical assistance.

### Conflict of interest statement

All the authors declare that they have no conflict of interest in this work.
